# Fully automatic tumor segmentation of breast ultrasound images with deep learning

**DOI:** 10.1002/acm2.13863

**Published:** 2022-12-09

**Authors:** Shuai Zhang, Mei Liao, Jing Wang, Yongyi Zhu, Yanling Zhang, Jian Zhang, Rongqin Zheng, Linyang Lv, Dejiang Zhu, Hao Chen, Wei Wang

**Affiliations:** ^1^ Collaborative Innovation Center of Advanced Microstructures School of Physics Nanjing University Nanjing China; ^2^ Department of Ultrasound Third Affiliated Hospital Sun Yat‐sen University Guangzhou China; ^3^ Department of Radiation Oncology Emory University Atlanta Georgia USA; ^4^ Institute for Brain Sciences Nanjing University Nanjing China; ^5^ Precision Care technology Hangzhou China

**Keywords:** automatic segmentation, breast cancer, breast ultrasound, deep learning

## Abstract

**Background:**

Breast ultrasound (BUS) imaging is one of the most prevalent approaches for the detection of breast cancers. Tumor segmentation of BUS images can facilitate doctors in localizing tumors and is a necessary step for computer‐aided diagnosis systems. While the majority of clinical BUS scans are normal ones without tumors, segmentation approaches such as U‐Net often predict mass regions for these images. Such false‐positive problem becomes serious if a fully automatic artificial intelligence system is used for routine screening.

**Methods:**

In this study, we proposed a novel model which is more suitable for routine BUS screening. The model contains a classification branch that determines whether the image is normal or with tumors, and a segmentation branch that outlines tumors. Two branches share the same encoder network. We also built a new dataset that contains 1600 BUS images from 625 patients for training and a testing dataset with 130 images from 120 patients for testing. The dataset is the largest one with pixel‐wise masks manually segmented by experienced radiologists. Our code is available at https://github.com/szhangNJU/BUS_segmentation.

**Results:**

The area under the receiver operating characteristic curve (AUC) for classifying images into normal/abnormal categories was 0.991. The dice similarity coefficient (DSC) for segmentation of mass regions was 0.898, better than the state‐of‐the‐art models. Testing on an external dataset gave a similar performance, demonstrating a good transferability of our model. Moreover, we simulated the use of the model in actual clinic practice by processing videos recorded during BUS scans; the model gave very low false‐positive predictions on normal images without sacrificing sensitivities for images with tumors.

**Conclusions:**

Our model achieved better segmentation performance than the state‐of‐the‐art models and showed a good transferability on an external test set. The proposed deep learning architecture holds potential for use in fully automatic BUS health screening.

## 1 INTRODUCTION

Breast cancer is the most common cancer in women and the second leading cause of death in women. According to the National Center for Health Statistics in the USA, breast cancer alone accounts for 30% of female cancers.^1^ Early detection through screening can greatly reduce the mortality and treatment costs of breast cancer.^2^, ^3^ Nowadays, ultrasonography has become one of the most prevalent approaches for the clinical detection of breast cancer due to its inexpensive, noninvasive, nonradioactive, and real‐time advantages.^4^, ^5^ Computer‐aided diagnosis (CAD) systems based on B‐mode breast ultrasound (BUS) have been developed to overcome the inter‐ and intra‐variabilities of the radiologists’ diagnoses and have demonstrated their ability to produce better clinical evaluations.^6^, ^7^

Image segmentation aims to mark the abnormal regions from the background. It is an essential step for CAD systems, and the quality of segmentation affects the diagnostic accuracy of the systems significantly,^8^ since many features, such as shape, aspect ratio, and smoothness of boundary, are related to the tumor contour and thus the segmentation result. Moreover, a real‐time and automatic segmentation system may assist radiologists in identifying tumors and provide a signal in case of human error.

With the development of deep learning techniques, automatic image segmentation approaches based on deep learning have been introduced to medical imaging and show significant improvements over conventional techniques.^9^, ^10^, ^11^, ^12^ Recently, several approaches based on deep learning have been developed for automatic BUS segmentation.^13^, ^14^, ^15^, ^16^, ^17^, ^18^, ^19^, ^20^, ^21^, ^22^ Yap et al.^13^ evaluated three deep learning approaches (Patch‐based LeNet, U‐Net, and Transfer Learning FCN‐AlexNet) for BUS mass detection on two different datasets and demonstrated an overall improvement by the deep learning approaches. Xian et al.^14^ published a B‐mode BUS image segmentation dataset with 562 images and compared the performance of five BUS segmentation methods quantitatively. A multi U‐Net algorithm was proposed to segment suspicious breast masses on US imaging in a previous study.^16^ The evaluation result on 258 patients revealed significant improvement over the original U‐Net. Zhuang et al.^18^ proposed an RDAU‐NET model based on the U‐Net structure to segment the masses in BUS images. They used dilated residual blocks and attention gates to replace basic blocks and skip connections in the U‐Net, respectively. Feng et al.^23^ proposed CPFNet, which combined two pyramidal modules to fuse global/multi‐scale context information; they tested the model on skin lesion, retinal linear lesion, thoracic organs, and retinal edema lesion segmentation tasks. Recently, Tang et al.^24^ presented a feature pyramid nonlocal network (FPNN) with transform modal ensemble learning for tumor segmentations in ultrasound images; the model was trained and evaluated on two public datasets, including 780 and 276 images, respectively.

**FIGURE 1:**
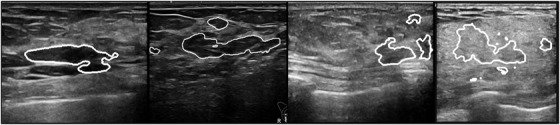
Examples of segmentation given by a DenseU‐Net if fed by normal BUS images (no masses are actually present). The area in the white line is the tumor region predicted by the model,which shows high false‐positive predictions. The network and the training details are given in the Supplementary

Although previous studies have shown an immense capacity for BUS segmentation, we believe that the reported approaches still have problems when used in actual clinical practice. The reasons are as follows. First, even for a breast with masses, the mass volume is much less than the total; thus, in a clinical screening, most scanned slices are normal, i.e., no tumors are present. Furthermore, the majority of breasts are normal in routine health screening.^25^ Second, automated models are expected to recognize these normal images and provide proper care, while most have not drawn enough attention on this issue. Third, nowadays, classification or segmentation systems usually rely on manually selected positive images with tumors, and lend less importance to the effect of normal images upon training models. For a fully automatic artificial intelligence (AI) system in the future, the above issues must be properly treated. Examples of the described issue are given in Figure 1, which shows the output of a DenseU‐Net model^26^ trained following usual procedures in literatures (detailed in the Supplementary). Note that the model gives many false‐positive segmentations when fed by normal images. These false‐positive outputs may discourage radiologists who use such systems as assistant and deteriorate the reliability of the AI system.

Another problem is the lack of labeled data for training AI systems, since it is time‐consuming and labor‐intensive for experienced radiologists to outline tumors.^5^ To the best of our knowledge, BUSI^27^ is the largest dataset for segmentation tasks that have been released to the public so far, which contains 780 BUS images, of which 133 are normal, 437 are benign, and 210 are malignant.

In this study, we compiled a new dataset that contains 1600 BUS images from 625 patients and a testing set that contains 130 BUS images from 120 patients. All images were manually segmented by experienced radiologists. To the best of our knowledge, this is the largest BUS dataset with pixel‐wise annotations. We designed and optimized a neural network with a new classification branch augmented to the segmentation net to address the problems described above. Then, we trained and tested the network and evaluated its performance on the classification of normal/abnormal images and on the segmentation of masses. Comparisons with several state‐of‐the‐art models were also made. We also optimized the network with different combinations of training/testing datasets, and studied its performance on benign and malignant tumors, respectively. Moreover, we performed ablation experiments to demonstrate the key role of the classification branch. Finally, we simulated the use of the model in actual clinic practice by processing videos recorded during BUS scans. The model gave very low false‐positive predictions on normal images without sacrificing sensitivities for images with tumors.

The major contributions of this study are summarized as follows: (1) compiled the largest breast US dataset with pixel‐wise annotations; (2) added a classification branch to the segmentation model and significantly decreased false positives particularly for normal images; (3) achieved better results than the state‐of‐the‐art models on both our test dataset and an external test set, demonstrating a good transferability; (4) simulated the use of the model in clinical practice and proved it is suitable for such a purpose.

## 2 MATERIALS AND METHODS

### 2.1 Datasets

**FIGURE 2:**
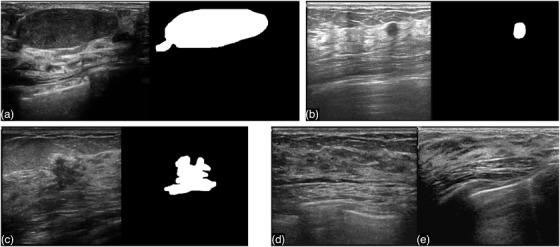
Image examples from our dataset. (a), (b), and (c) are images with masses (the segmentation is delineated manually by radiologists); (d) and (e) are normal images (without masses)

We built a database that contains 1600 BUS images in clinical breast examinations conducted in the Third Affiliated Hospital of Sun Yat‐sen University (referred to as SYUSI) from 2017 to 2021 for 625 women aged 20–85 years. The negative, benign, and malignant cases are 212, 217, and 196, respectively. Of the 1600 BUS images, 405 were images with benign masses, 372 with malignant tumors, and 823 were normal (without masses). The contours of masses were delineated and confirmed by experienced radiologists (M.L., Y.Z., Y.Z., R.Z.). The normal images were also visually inspected and confirmed by the same team. The average image size is 700 × 500 pixels. The information is summarized in Table 1, and some examples are shown in Figure 2.

**TABLE 1:** Overview of the datasets used in this study. The SYUSI and SY‐test datasets were compiled for this study. The BUSI dataset was from the Baheya Hospital, Egypt, and ST‐test was from the First Affiliated Hospital of Shantou University. The second column indicates whether the dataset was used for training, validation, or testing. The fifth column gives the number of normal cases (no masses are present). The last column shows the number of cases with masses and the number of benign and malignant findings within brackets, provided that such classification exists

Dataset	Usage	Level	Total	Normal	Cases with masses (benign/malignant)
SYUSI	Train/validation	Image	1600	823	777 (405/372)
		Patient	625	212	413 (217/196)
BUSI	Train/validation	Image	780	133	647 (437/210)
		Patient	600	–	–
SY‐test	Test	Image	130	50	80 (40/40)
		Patient	120	40	80 (40/40)
ST‐test	Test	Image	42	0	42
		Patient	–	–	–

To improve the heterogeneity of data and generalization ability of the trained model, we also combined the BUSI dataset with ours to train the model. The BUSI dataset was collected from Baheya Hospital, Egypt.^27^ BUS images were obtained from 600 female patients aged 25–75 years. The dataset included 780 images, of which 133 showed normal, 437 benign, and 210 malignant findings. The average image size was 500 × 500 pixels. We used the above two datasets to train our model.

To evaluate the model performance, we included 120 patients – the negative, benign, and malignant cases are 40, 40, and 40, respectively, from Third Affiliated Hospital of Sun Yat‐sen University and build a testing dataset (referred to as SY‐test). SY‐test contains 130 BUS images: 40 images were with malignant masses, 40 with benign masses, and 50 without masses. All the masses were proved by biopsy or surgery results. For the 80 images with masses, freehand segmentations were established by senior radiologists (M.L., Y.Z., Y.Z.). We also tested the model on 42 BUS images from the Imaging Department of the First Affiliated Hospital of Shantou University (ST‐test) for additional tests,^28^ which were available in a previous study.^18^ Note that ST‐test was from an external facility and thus posed a significantly challenge to the model. All the relevant datasets are summarized in Table 1.

### 2.2 Architecture and logic of the neural network model

**FIGURE 3:**
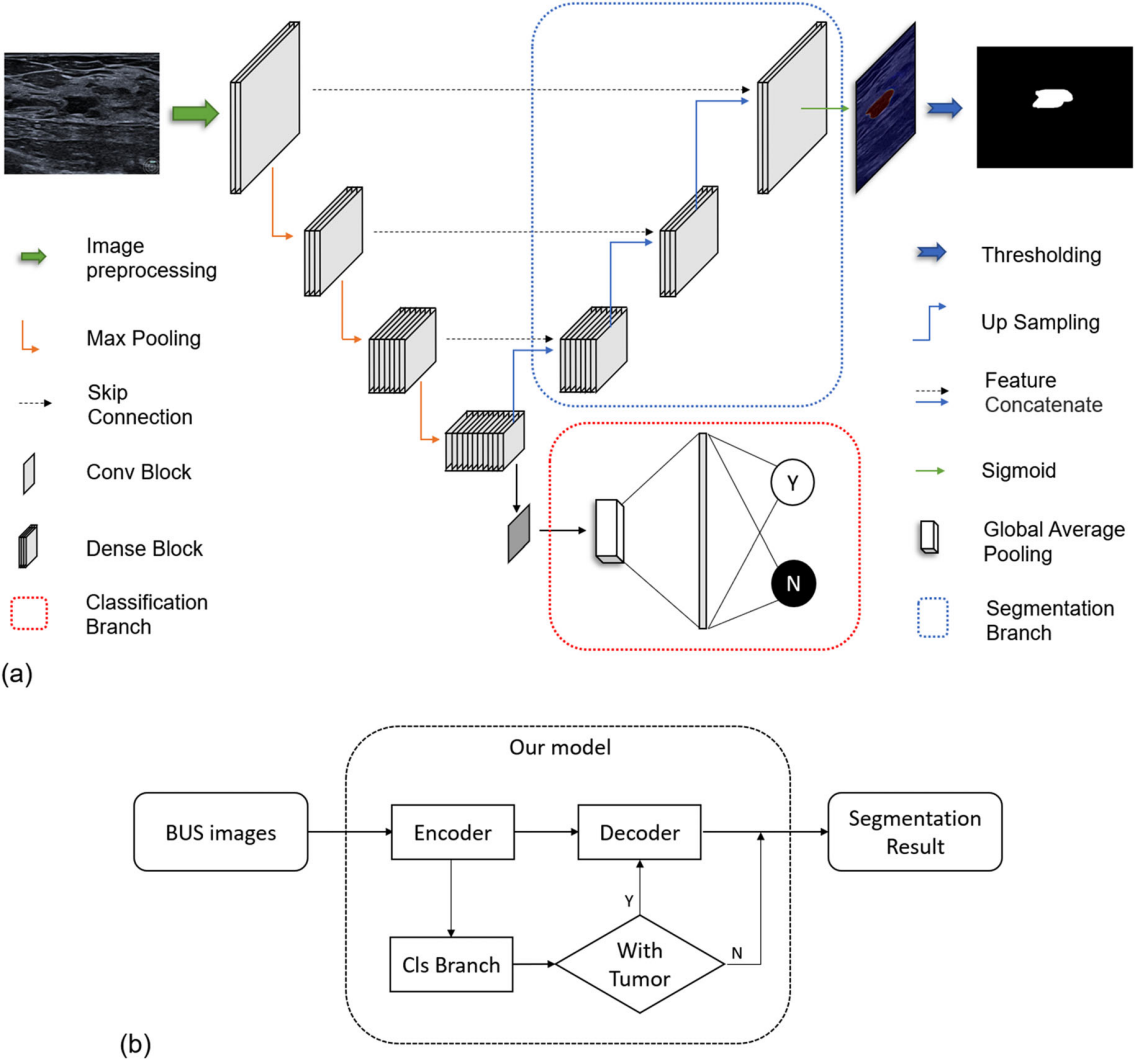
(a) The proposed architecture of our network. It contains a classification branch (lower right) and a segmentation branch (upper right). The input to the model is a BUS image, and the outputs are the category of the image given by the classification branch (normal or abnormal) and the pixel‐wise segmentation given by the segmentation branch if the image is abnormal. (b) Flowchart showing the logic of the network

The network is composed of two branches, a classification branch and a segmentation branch, as depicted in Figure 3. The two branches share the encoder layers. The segmentation part of the network is based on the U‐Net^29^ structure with DenseNet^30^ as the backbone. The network is U‐shaped, mainly composed of an encoder, a decoder, and skip connections. The encoder reduces the spatial dimension (feature scale) with max pooling and extracts context features. The decoder recovers the spatial dimension with up‐sampling and propagates context information to higher resolution layers. The skip connection transfers the features in the encoder directly to the decoder of the same scale to recover possible information loss. The features in the skip connection and up‐sampling in the decoder are concatenated together and propagated to the next layer. In detail, the encoder contains four dense blocks and three pooling layers, and the decoder contains three dense blocks and three up‐sampling layers. Dense blocks are composed of several densely connected conv blocks, which contain two batch normalization layers and two convolutional layers with kernel size of 1 and 3, respectively. Four dense blocks in the encoder are composed of 3, 4, 8, and 12 conv blocks, respectively. Three dense blocks in the decoder are composed of 8, 4, and 3 conv blocks, respectively.

One notable new feature of the model is the added classification branch shown in Figure 3a (bottom right). The classification branch is connected to the last layer of the encoder and is composed of a convolution block, a global average pooling layer, and a fully connected layer. It was designed to classify the input images into normal/abnormal and prevent normal images entering the following decoder. It significantly increased the performance of the model, as will be discussed later.

### 2.3 Training strategies

The SYUSI and BUSI datasets were combined to train the model to increase generalization ability of the model. All images, including both normal and abnormal ones, were fed to the network to optimize the parameters. Before fed to the network, the images were resized to 256 × 256, and the pixel values were normalized to [−1, 1]. The images were also subjected to random horizontal flipping for data augmentation.

Multi‐task loss including classification loss and segmentation loss was employed to optimize our model. Classification loss was the binary cross‐entropy loss function, and segmentation loss was a combination of pixel‐wise binary cross‐entropy loss function^31^ and dice loss function.^32^ The combination of binary cross‐entropy loss and dice loss can alleviate the imbalance of foreground and background in segmentation cases and make the training process more stable.^33^ The segmentation loss function on a single image is defined as follows:

(1) $$\begin{array}{c} L_{\mathrm{BCE}}\left(y,t\right)=-\sum_{\mathrm{ij}}\left(\mathrm{tlog}\left(y\right)+\left(1-t\right)\log\left(1-y\right)\right),\\ L_{\mathrm{Dice}}\left(y,t\right)=1-\frac{2\sum_{\mathrm{ij}}\mathrm{yt}+1}{\sum_{\mathrm{ij}}y+\sum_{\mathrm{ij}}t+1},\\ \;\;\;L_{\mathrm{seg}}=L_{\mathrm{BCE}}+L_{\mathrm{Dice}} \end{array}$$

Here, *t* is the ground truth and *y* is the predicted outcome. *i* and *j* represent a pixel on the image.

During training, 10‐fold validation was employed: (1) images in the training datasets were divided into ten folds randomly; (2) nine folds were used for training and one was left for validation; (3) the model with highest validation dice similarity coefficient was selected as the final model; (4) the statistics for average and standard deviation results were calculated across ten experiments. An adaptive moment estimation (Adam)^34^ optimizer with an initial learning rate of 0.001 was used to train the model.

### 2.4 Evaluation metrics

Our model had two outputs, classification categories and segmentation marks. We evaluated the classification results using the area under the receiver operating characteristic curve (AUC), sensitivity, specificity, accuracy, and F1‐score. As for the segmentation result, both area error metrics and boundary error metrics were utilized. The area error metrics include the dice similarity coefficient (DSC), Jaccard index (JI), true‐positive ratio (TPR), false‐positive ratio (FPR),^14^ and false‐negative ratio (FNR).^35^

(2) $$\begin{array}{ccc} \mathrm{DSC} & = & \frac{1+2\left|A_{m}\cap A_{r}\right|}{1+\left|A_{m}\right|+\left|A_{r}\right|},\\ \mathrm{JI} & = & \frac{\left|A_{m}\cap A_{r}\right|}{\left|A_{m}\cup A_{r}\right|},\\ \mathrm{TPR} & = & \frac{\left|A_{m}\cap A_{r}\right|}{\left|A_{m}\right|},\\ \mathrm{FPR} & = & \frac{\left|A_{m}\cup A_{r}-A_{m}\right|}{\left|A_{m}\right|},\\ \mathrm{FNR} & = & \frac{\left|A_{m}\cup A_{r}-A_{r}\right|}{\left|A_{m}\right|}. \end{array}$$

In the equations, $A_{m}$ is the pixel set of the mass region of ground truth mass and $A_{r}$ is the pixel set of the mass region generated by the segmentation method. Further, two boundary error metrics, namely, Hausdorff error (HE) and mean absolute error (MAE),^36^, ^37^ are applied to measure the worst possible disagreement and the average disagreement between two contours, respectively. All segmentation metrics (except DSC) are only well‐defined on images with tumors.

The metric DSC is a generalized version to the one in literatures to cover normal images as well. For normal images, $A_{m}=\;0$, $\mathrm{DSC}\;=\frac{1}{1+A_{r}}\;$. Therefore, DSC will be 1 if the $A_{r}$ is correctly predicted to be zero. It will be smaller than 1 if any non‐zero segmented area is predicted for a normal image.

### 2.5 Model comparison approaches

The performance of our approach was compared to several state‐of‐the‐art segmentation models, including U‐Net,^29^ UNet++,^38^ CE‐Net,^39^ CPFNet,^23^ and FPNN.^24^ In addition, ablation experiments were carried out to explore the role of the classification branch. The baseline model is a U‐Net with DenseNet as backbone, which is similar to our model but with the classification branch deleted. The baseline model is referred to as Model‐2. All these models were trained and tested on the aforementioned datasets in the same way as in our model. For further tests, Model‐2 was trained with only positive images (with tumors) in datasets, and the resulted model was referred to as Model‐2‐pos. It is worth mentioning that this particular way of training Model‐2‐pos is commonly used in tumor segmentation studies.

In order to better visualize the improvement of our model against general segmentation models (such as Model‐2‐pos) in clinical application, we simulated the use of two models on routine BUS screening. Specifically, the radiologists in our team recorded videos when they performed BUS scans for patients. The videos started 1–2 s before the masses appeared and ended after the mass was out of the detecting region of the probe. The videos usually last for 7–8 s. We extracted all frames from the videos and use the model to segment them individually. The segmentation results are recorded in videos in sequence.

## 3 RESULTS

### 3.1 Ability to distinguish images with and without masses

Our network model was designed to first classify an input BUS image to a normal or an abnormal category and then segment it if judged to be abnormal (i.e., masses are present). The logic is shown in Figure 3b. In this section, we report the classification performance of our model, as shown in Table 2. For the first classification task, the AUC, sensitivity, specificity, accuracy, and F1‐score on the SY‐test dataset were 0.991, 0.950, 1.000, 0.969, and 0.974, respectively, showing that our model has an extremely low false‐positive rate in discriminating normal/abnormal images. For a comparison, two radiologists in our team carried out the same classification task on the SY‐test dataset. The mean sensitivity, specificity, accuracy, and F1‐score were 0.969, 0.950, 0.962, 0.969, respectively. AUC cannot be calculated since binary results instead of probabilities were given by human experts. Our model outperformed human experts on specificity, accuracy and F1‐score.

**TABLE 2:** Classification performance of human experts and our model on the SY‐test datasets. The classification branch of the model classifies the input images into two categories, normal (without tumor present) versus abnormal (with tumor present)

	AUC	Sensitivity	Specificity	Accuracy	F1
Experts	_	0.969	0.950	0.962	0.969
Our model	0.991	0.950	1.000	0.969	0.974

### 3.2 Performance of mass segmentation

Table 3 shows the segmentation results of eight different models. It can be seen that the first six ones, including U‐Net, UNet++, CE‐Net, CPFNet, FPNN and Model‐2, show similar metrics, while our model is significantly better than these six models on all metrics. For example, the DSCs of the first six models range from 0.743 to 0.826, while ours is 0.898; the FPRs for the first six range from 0.150 to 0.296, while ours is significantly low (0.097). The comparison with Model‐2‐pos will be discussed later.

**TABLE 3:** Comparison of segmentation results on the SY‐test dataset (Mean ± Standard Deviation)

Model	Area metrics					Boundary metrics	
	DSC	JI	TPR	FPR	FNR	MAE	HE
U‐Net^29^	0.746 ± 0.015	0.644 ± 0.012	0.789 ± 0.020	0.296 ± 0.026	0.211 ± 0.020	11.492 ± 0.745	43.842 ± 2.090
UNet++^38^	0.743 ± 0.014	0.642 ± 0.016	0.762 ± 0.021	0.216 ± 0.036	0.238 ± 0.021	10.885 ± 0.878	41.079 ± 2.171
CE‐Net^39^	0.796 ± 0.018	0.670 ± 0.014	0.785 ± 0.008	0.220 ± 0.049	0.215 ± 0.008	8.260 ± 0.630	35.393 ± 2.164
CPFNet^23^	0.804 ± 0.011	0.694 ± 0.009	0.800 ± 0.011	0.177 ± 0.027	0.200 ± 0.011	7.781 ± 0.576	32.684 ± 2.417
FPNN^24^	0.805 ± 0.017	0.672 ± 0.014	0.767 ± 0.016	0.181 ± 0.014	0.233 ± 0.016	9.967 ± 0.741	39.793 ± 1.989
Model‐2	0.826 ± 0.013	0.698 ± 0.011	0.789 ± 0.016	0.150 ± 0.041	0.211 ± 0.008	7.430 ± 0.709	31.462 ± 1.631
Model‐2‐pos	0.528 ± 0.005	0.787 ± 0.007	0.855 ± 0.006	0.097 ± 0.013	0.145 ± 0.006	5.726 ± 0.394	27.061 ± 1.446
Our model	**0.898 ± 0.015**	**0.791 ± 0.007**	**0.859 ± 0.008**	**0.097 ± 0.018**	**0.141 ± 0.008**	**5.708 ± 0.618**	**26.896 ± 1.593**

The best results among all models are shown in bold.

Table 4 illustrates the segmentation results of all models on an external ST‐test dataset. Our model also shows the best results on all metrics. The ranks of the models are consistent with those in Table 3. Considering that the ST‐test dataset was from another facility and kept invisible during training, the transferability of our model proved to be good.

**TABLE 4:** Comparison of segmentation results on an external ST‐test dataset (Mean ± Standard Deviation). The best results among all models are shown in bold

Model	Area metrics					Boundary metrics	
	DSC	JI	TPR	FPR	FNR	MAE	HE
U‐Net^29^	0.825 ± 0.011	0.747 ± 0.013	0.870 ± 0.018	0.188 ± 0.035	0.130 ± 0.018	7.115 ± 0.489	32.705 ± 2.459
UNet++^38^	0.834 ± 0.005	0.750 ± 0.007	0.868 ± 0.007	0.165 ± 0.012	0.132 ± 0.007	6.979 ± 0.344	30.09 ± 1.288
CE‐Net^39^	0.834 ± 0.016	0.774 ± 0.017	0.897 ± 0.011	0.173 ± 0.045	0.103 ± 0.011	5.832 ± 0.535	26.676 ± 2.697
CPFNet^23^	0.850 ± 0.008	0.791 ± 0.007	0.899 ± 0.008	0.142 ± 0.012	0.101 ± 0.008	5.290 ± 0.196	24.224 ± 0.850
FPNN^24^	0.846 ± 0.007	0.777 ± 0.007	0.884 ± 0.005	0.159 ± 0.011	0.116 ± 0.005	6.172 ± 0.243	29.136 ± 1.063
Model‐2	0.831 ± 0.012	0.780 ± 0.013	0.905 ± 0.008	0.179 ± 0.032	0.095 ± 0.008	5.570 ± 0.371	26.029 ± 1.665
Model‐2‐pos	0.888 ± 0.002	0.827 ± 0.003	0.905 ± 0.003	0.098 ± 0.006	0.095 ± 0.003	4.321 ± 0.119	21.705 ± 0.841
Our model	**0.890 ± 0.002**	**0.830 ± 0.004**	**0.906 ± 0.005**	**0.096 ± 0.006**	**0.094 ± 0.005**	**4.189 ± 0.121**	**21.071 ± 0.610**

**FIGURE 4:**
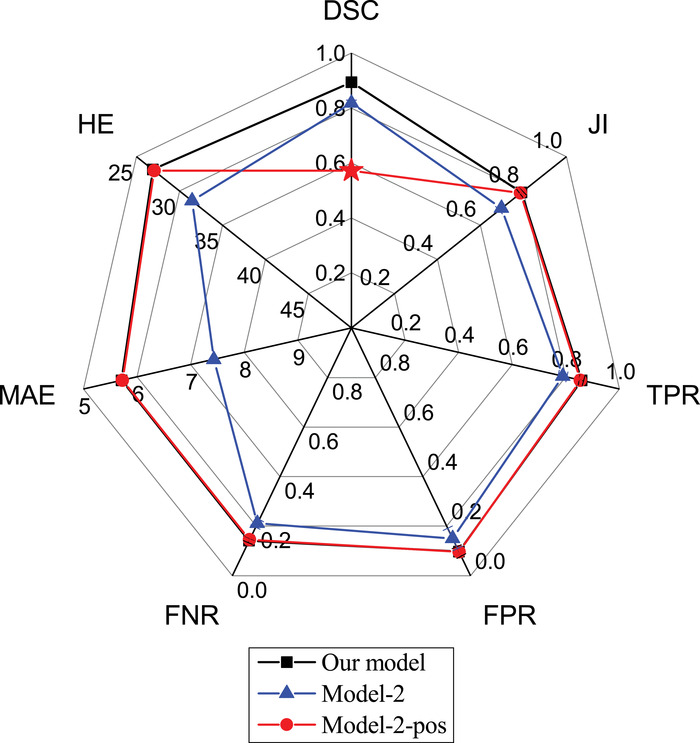
Segmentation performance of our model compared with Model‐2 and Model‐2‐pos. The architecture of the latter two is a U‐Net with DenseNet as backbone, similar to our model but with the classification branch deleted. Model‐2 was trained using the same training dataset as our model, whereas Model‐2‐pos was trained using only the positive images in the same dataset

In Figure 4, we show the results of an ablation experiment that compares the proposed model with Model‐2 and Model‐2‐pos on the SY‐test dataset. The calculated metrics are detailed in Table 3. It can be seen that Model‐2 yields significantly worse performance in terms of both the area error metrics (DSC, JI, TPR, FPR, and FNR) and boundary error metrics (MAE and HE). We checked its outputs and found that the deteriorated performance was mostly attributed to normal images, which were given wrong segmentation masks even if no masses are present (false positive issues). Based on the comparison, we argue that normal images acted like “noise” to the segmentation network if directly fed to Model‐2. By contrast, the added classification branch filtered these “noises” and increased the overall performance.

To further prove the argument, Model‐2‐pos was trained with only the positive images (with masses) in the dataset. This setting was commonly seen in previous studies, where the models were designed, trained, and validated only on positive BUS images. The result is shown by the red line in Figure 4. In this setting, Model‐2‐pos (red line) gives almost the same results as our model (black line) on all metrics except DSC (red star). The extremely low value of DSC is again caused by false‐positively predicted regions frequently observed in normal images. Examples can be found in Figure 1 and more in the Supplementary Material. The metrics other than DSC are defined only on positive images and hence cannot reflect model performance on normal images. In short, Model‐2 either gives a low performance on all metrics (blue line in Figure 4) or a significantly low DSC (red star in the red line), depending on how the model was trained. By contrast, our model solved this problem.

Previous, Shareef et al.^40^ developed an Enhanced Small Tumor‐Aware network (ESTAN) and tested it on three datasets. The DSCs of their model ranged from 0.78 to 0.92, TPR ranged from 0.80 to 0.91, FPR from 0.36 to 0.07, depending on different datasets used for training and testing. However, a rigorous comparison of models is only possible with a public competition that tests models on its internal dataset that is blinded to all participators.

### 3.3 Mass segmentation tested with different tumors

Table 5 presents the respective performance of the model on benign and malignant tumors. The results of the benign tumors are better. This is attributed to the heterogeneous feature of malignant tumors with respect to shape, size, border roughness, echodensity, posterior acoustic shadow, etc.^41^

**TABLE 5:** Segmentation results of our model on benign and malignant cases in the SY‐test dataset

Data	Area metrics					Boundary metrics	
	DSC	JI	TPR	FPR	FNR	MAE	HE
Benign	0.893	0.831	0.886	0.075	0.104	3.682	15.900
Malignant	0.829	0.751	0.832	0.119	0.178	7.734	37.892

**FIGURE 5:**
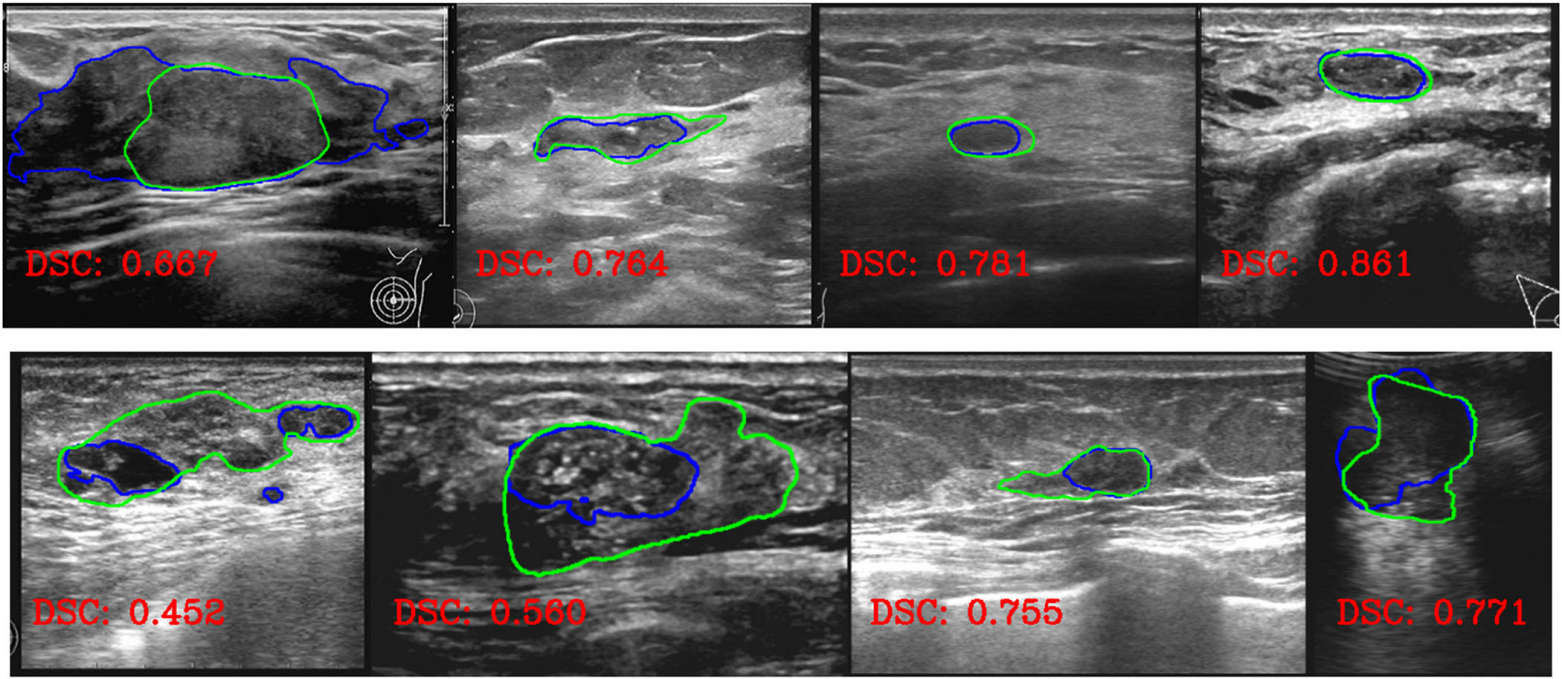
Four benign (upper) and four malignant (bottom) tumors with the lowest DSCs in the SY‐test dataset. Green lines show the ground truth delineated by radiologists, while blue lines show the predicted ones by our model

Figure 5 presents cases with the lowest DSCs selected from the SY‐test dataset. They represent the most difficult cases for our model. For the benign cases in the upper row, the DSCs range from 0.67 to 0.86, and the overlap between the ground truth and prediction is generally good with a visual inspection. For the four malignant tumors in the bottom row, the DSCs range from 0.45 to 0.77. Although the DSC values appear to be unimpressing, the model still identified rough locations of the tumors. Therefore, we believe that our model is still useful in assisting radiologists to localize masses in these difficult cases.

### 3.4 Simulation of the use of the model on routine BUS screening

Since all segmentation models should be tested in clinical scenarios ultimately, we simulated the use of our model and Model‐2‐pos on routine BUS screening. The detailed procedures are described in the section Materials and Methods. For ease of presentation, here we show five snapshots from the recorded videos within a roughly uniform time interval. The extracted snapshots and segmentation results are given below. The whole videos can be downloaded at https://www.doi.org/10.6084/m9.figshare.19105910.

**FIGURE 6:**
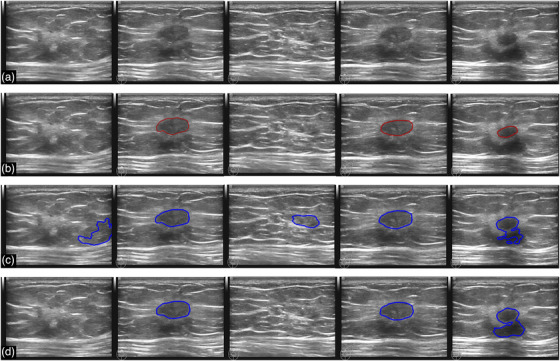
Segmentation results for a breast with invasive ductal carcinoma (malignant case). (a) The original images sequentially extracted from a video recorded during a BUS scan. (b) The tumor regions (ground truth) delineated by experienced radiologists. (c) and (d) are the segmentation results given by Model‐2‐pos (corresponding to the red line in Figure 4) and our model, respectively. The marked regions indicate the predicted tumors

Figure 6 shows five snapshots from a video of length 7 s, recorded for a patient who was diagnosed with an invasive ductal carcinoma, which is a malignant tumor. The condition was confirmed with both biopsy and surgery. From top down, the four rows present the original images, the tumor regions delineated by experienced radiologists (ground truth), the tumor regions predicted by Model‐2‐pos, and those by our model, respectively. For the first and third snapshots, Model‐2‐pos reported normal tissues as tumors and thus gave a false‐positive prediction. By contrast, our model can recognize them correctly. For the second, fourth, and fifth columns with tumors present, both models worked well and similarly.

**FIGURE 7:**
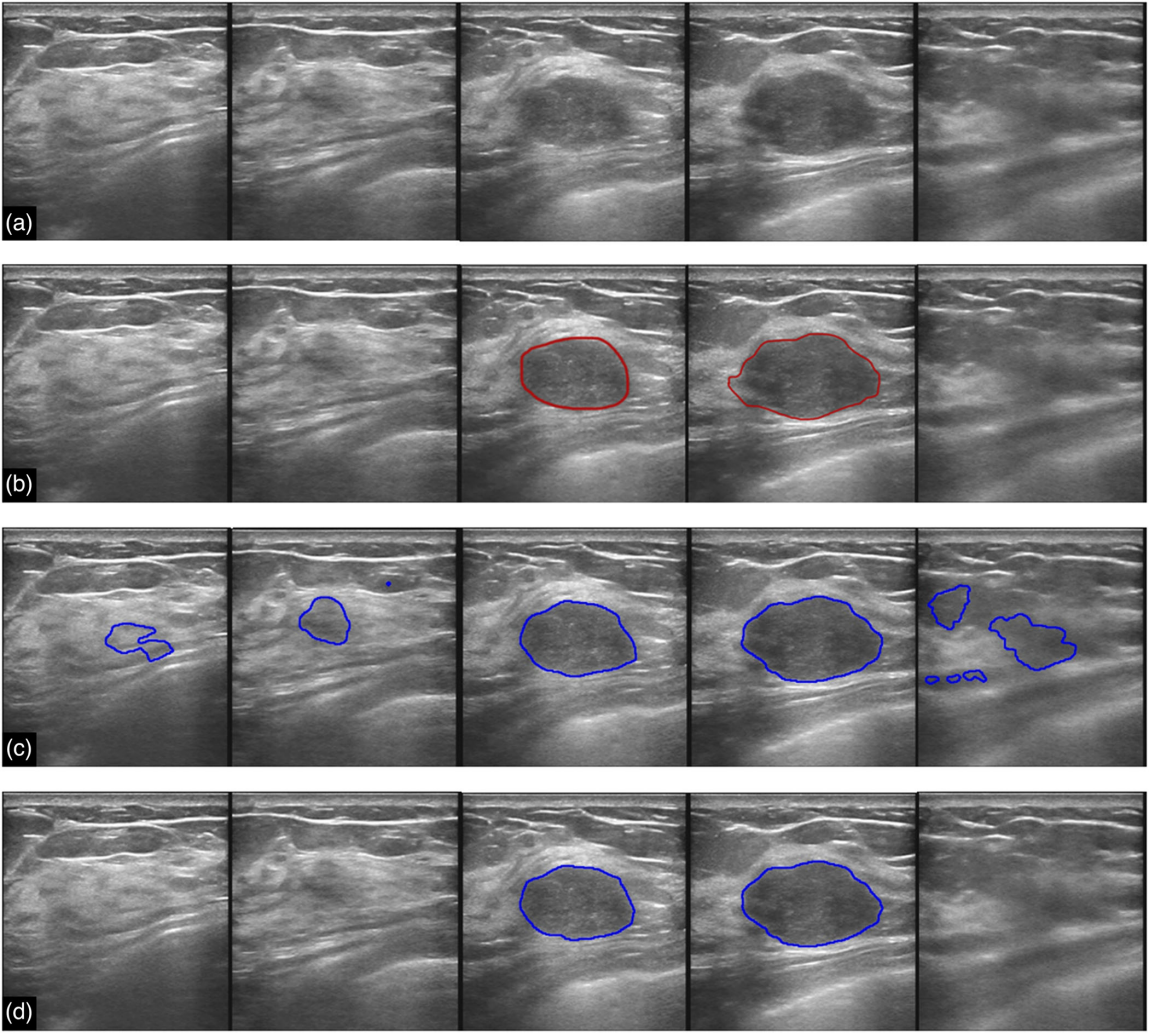
Segmentation results for a breast with fibroadenoma (benign case). (a) The original images sequentially extracted from a video recorded during a BUS scan. (b) The tumor regions (ground truth) delineated by experienced radiologists. (c) and (d) are the segmentation results given by Model‐2‐pos (corresponding to the red line in Figure 4) and our model, respectively. The marked regions indicate the predicted tumors

Figure 7 shows another breast with fibroadenoma, which is a benign mass. The condition was confirmed by biopsy. Model‐2‐pos gave a positive prediction for each snapshot, although no tumors are present in the first, second, and last snapshots. By contrast, our model recognized all snapshots correctly – no false‐positive predictions without sacrificing sensitivity.

Given the limited space, we only show two examples in the main text. More snapshots can be seen in the supplementary and more videos at https://www.doi.org/10.6084/m9.figshare.19105910.

## 4 DISCUSSION AND CONCLUSION

BUS serves as a commonly seen modality in clinical breast cancer screenings. While the majority of BUS scans are normal images without tumors, segmentation approaches such as U‐Net often predict mass regions for these cases. Such false‐positive problem becomes serious if a fully automatic artificial intelligence system is used for routine screening in the future. To address the problem, we built a new dataset containing 1600 BUS images from 625 patients, and developed a novel deep learning architecture consisting of a classification branch and a segmentation branch sharing the same encoder. For an input BUS image, the model first determines it is whether a normal image without masses or an abnormal with masses, and then outputs the segmentation result for the latter case.

We tested the model's competency to classify BUS images into normal or abnormal categories. The AUC, sensitivity, specificity, accuracy, and F1‐score on the SY‐test dataset were 0.991, 0.950, 1.000, 0.969, and 0.974, respectively. We then tested the segmentation performance of the model. The DSC was 0.898, and the false‐positive rate was 0.097 on the SY‐test dataset. The performance was even slightly better on an external testing dataset (ST‐test). The results proved a good transferability of our model to different datasets. Comparisons against U‐Net, UNet++, CE‐Net, CPFNet, and the most recent FPNN showed that our model exhibits the best performance on our testing dataset and the external testing dataset.

It is worth mentioning that the most recent (current version Nov 2021) FPNN developed a very sophisticated strategy by fusing multilevel features including long‐range dependencies and introducing a special form of ensemble learning, however, it still gave slightly worse performance than our model on two testing datasets. For example, the DSC on SY‐test given by FPNN is 0.805, while ours is 0.898; the FPR of FPNN is 0.181, while ours is 0.097 (Table 3). The results on ST‐test are similar (Table 4). Therefore, we argue that the key to reduce false positives and improve the performance is how to properly handle images without tumors. Our model was particularly designed for this issue and indeed improved the performance.

We did an ablation experiment for further supporting the above argument. We compared the performance of our model with those of Model‐2, which was similar to our model but with the classification branch deleted. Model‐2 manifested a significantly decreased performance (e.g., a decrease of 0.07 on DSC), since it often gave false‐positive predictions for normal images. If normal images were deliberately deleted from the training dataset, i.e., only positive images were used for training, the resulted Model‐2‐pos achieved a better performance on positive images but even worse performance on identifying normal images (e.g., DSC = 0.528). We also did another ablation experiment on U‐Net (described in the Supplementary Material). The results were similar to those of Model‐2, i.e., U‐Net either gave a low performance on all metrics or a very low DSC regardless of how the model was trained. Therefore, pure U‐Net architecture models do not fit the scenario in routine BUS screening, where the majority of BUS images in clinic are normal. By contrast, our model can treat both normal and abnormal images simultaneously without additional computational efforts by adding a classification branch and allowing the branch to share the encoder layers of the segmentation network. The proposed model indeed improved the performance, even comparing with the most recent state‐of‐the‐art models.

We believe that tumor segmentation models should be tested in actual clinical practice ultimately. For this purpose, and to temporarily bypass the technical complications of implementing our model in PACS systems in hospital, we simulated the use of the model in clinic screening by post‐processing videos recorded during BUS scans. The experiments showed that the model significantly reduced false positives in normal images without sacrificing the sensitivity for images with masses. We believe that our model could be a key module in a fully automatic AI system for routine BUS screening in the future.

This study has some limitations. First, the training dataset is still not large enough, which might limit the generalization performance of our model. Though we have tested it on some foreign sourced images, further tests of the model on large‐scale datasets, particularly on those from other facilities, are necessary for the application of the model in clinic practice. Second, the segmentation result is slightly worse for malignant tumors than for benign ones, apparently due to their larger heterogeneity in shape, border roughness, echodensity, and so on. Traditional radiomics features may be combined with deep learning features to solve for the problem.

## CONFLICTS OF INTEREST

No conflicts of interest.

## Supporting information

Supporting information
