# Supplementary material for: Fully automatic tumor segmentation of breast ultrasound images with deep learning
Source: J Appl Clin Med Phys. 2022 Dec 9;24(1):e13863. doi: 10.1002/acm2.13863 (PMC9859996; doi:10.1002/acm2.13863)
Supplement: Supplementary file 1 — Supporting information [file ACM2-24-e13863-s001.docx]

**Supplementary**

# Architecture and training procedures of Model-2

Model-2 described in the main text employed a U-Net architecture with DenseNet as backbone. The only difference between Model-2 and our proposed model is the absence of a classification branch in the former. A segmentation loss combining binary cross-entropy loss and dice loss was used. Adam optimizer with an initial learning rate of 0.001 was adopted to train the model.

Model-2 was trained with two different settings. The first one used the whole training dataset, which is the same as that used in our model. In this setting, negative (or referred to as normal) images (without tumors) are exposed to the segmentation model so that they can be recognized by the model. In the second setting, only positive (or referred to as abnormal) images (with tumors) were used for training, similar to the setting commonly used in previous studies. This setting corresponds to the Model-2-pos in the main text. Models trained with the latter setting usually have good performance on mass segmentation for positive images, however, tend to generate false positive masks for normal (negative) images.

# Segmentation results of our model, Model-2, Model-2-pos trained with different losses

Table S1 shows the segmentation results of our model, Model-2, Molde-2-pos training with binary cross-entropy (BCE) loss, dice loss, and the combination of both, respectively. For our model and Model-2-pos, the loss function had only a slight impact (about 0.01 for most area error metrics and 0.5 to 1 for boundary error metrics). For Model-2, the binary-cross entropy loss got a relatively low DSC, indicating that the pixel-wise binary-cross entropy loss does not perform well in normal images. Compared with using cross-entropy loss or dice loss alone, the combination loss gave slightly better segmentation results for both our model and Model-2-pos, and gave balanced results for Model-2.

**Table S1**. Segmentation results of our model, Model-2, Molde-2-pos trained with binary cross-entropy (BCE) loss, dice loss and the combination of both, respectively (Mean ± Standard Deviation). The best results are shown in bold.

| **Model** | **Loss** | **Area error metrics** | | | | | **Boundary error metrics** | |
| --- | --- | --- | --- | --- | --- | --- | --- | --- |
|  |  | **DSC** | **JI** | **TPR** | **FPR** | **FNR** | **MAE** | **HE** |
| Our  model | BCE | 0.886  ±0.016 | 0.772  ±0.008 | 0.845  ±0.011 | 0.133  ±0.035 | 0.154  ±0.011 | 6.005  ±0.473 | 27.710  ±1.969 |
|  | Dice | **0.900**  **±0.018** | 0.782  ±0.007 | 0.847  ±0.009 | **0.093**  **±0.015** | 0.153  ±0.009 | 6.004  ±0.412 | 27.884  ±1.326 |
|  | Combined | 0.898  ±0.015 | **0.791**  **±0.007** | **0.859**  **±0.008** | 0.097  ±0.018 | **0.141**  **±0.008** | **5.708**  **±0.618** | **26.896**  **±1.593** |
| Model-2 | BCE | 0.575  ±0.044 | **0.723**  **±0.014** | 0.773  ±0.013 | **0.094**  **±0.015** | 0.227  ±0.013 | **6.122**  **±0.431** | **28.691**  **±1.464** |
|  | Dice | **0.841**  **±0.014** | 0.702  ±0.013 | 0.782  ±0.014 | 0.134  ±0.026 | 0.218  ±0.014 | 7.443  ±0.571 | 32.765  ±2.008 |
|  | Combined | 0.826  ±0.013 | 0.698  ±0.011 | **0.789**  **±0.016** | 0.150  ±0.041 | **0.211**  **±0.008** | 7.430  ±0.709 | 31.462  ±1.631 |
| Model-2-pos | BCE | 0.522  ±0.008 | 0.773  ±0.007 | 0.838  ±0.005 | 0.117  ±0.021 | 0.162  ±0.005 | 5.981  ±0.178 | 27.776  ±1.516 |
|  | Dice | 0.518  ±0.022 | 0.770  ±0.007 | 0.838  ±0.006 | 0.133  ±0.030 | 0.162  ±0.006 | 6.401  ±0.436 | 28.873  ±1.580 |
|  | Combined | **0.528**  **±0.005** | **0.787**  **±0.007** | **0.855**  **±0.006** | **0.097**  **±0.013** | **0.145**  **±0.006** | **5.726**  **±0.394** | **27.061**  **±1.446** |

# Segmentation results of our model trained with SYUSI and BUSI, respectively

Table S2 shows the segmentation results of our model trained with SYUSI and BUSI, respectively. The two models are referred to as the first model and the second model, respectively. Judged by most metrics, the first model is far better than the second one. The TPR and FNR of the second model, however, are slightly better than the first, but at the cost of a large FPR of more than twice.

**Table S2**. Segmentation results of our model trained with SYUSI and BUSI, respectively (Mean ± Standard Deviation). The best results are shown in bold.

| **Test set** | **Training set** | **Area error metrics** | | | | | **Boundary error metrics** | |
| --- | --- | --- | --- | --- | --- | --- | --- | --- |
|  |  | **DSC** | **JI** | **TPR** | **FPR** | **FNR** | **MAE** | **HE** |
| SY-test | SYUSI | **0.900**  **±0.008** | **0.778**  **±0.007** | 0.845  ±0.008 | **0.115**  **±0.032** | 0.155  ±0.008 | **6.111**  **±0.457** | **28.025**  **±1.871** |
|  | BUSI | 0.787  ±0.011 | 0.706  ±0.012 | **0.850**  **±0.018** | 0.253  ±0.030 | **0.150**  **±0.018** | 9.420  ±0.821 | 38.536  ±1.990 |
| ST-test | SYUSI | **0.889**  **±0.002** | **0.827**  **±0.004** | 0.899  ±0.005 | **0.094**  **±0.009** | 0.101  ±0.005 | **4.310**  **±0.178** | **21.738**  **±0.969** |
|  | BUSI | 0.851  ±0.007 | 0.780  ±0.011 | **0.929**  **±0.008** | 0.208  ±0.025 | **0.071**  **±0.008** | 5.805  ±0.412 | 24.918  ±1.743 |

# Ablation study on U-Net

To further explore the roles of the classification branch, we constructed three more models. The first is U-Net with a classification branch. The second is U-Net. The third is U-Net trained with only positive images. We trained the first two on the whole dataset, and trained the third on the dataset with only positive images. The resulted models are referred as to UNet-cls, UNet and UNet-pos, respectively. They are similar to our proposed model, Model-2, and Model-2-pos, respectively, while the backbone modules are different. We show the segmentation results in Table S3. It can be seen that, 1) our model achieved the best performance on all metrics; 2) UNet-cls with a classification branch performed better than those without it. The results are consistent with those given in the main text.

**Table S3**. Segmentation results of our model, Model-2, Model-2-pos, UNet-cls, UNet, and UNet-pos, respectively (Mean ± Standard Deviation). The first column shows the corresponding test datasets. The best results are shown in bold.

| **Test set** | **Model** | **Area error metrics** | | | | | **Boundary error metrics** | |
| --- | --- | --- | --- | --- | --- | --- | --- | --- |
|  |  | **DSC** | **JI** | **TPR** | **FPR** | **FNR** | **MAE** | **HE** |
| SY-test | Our model | **0.898**  **±0.015** | **0.791**  **±0.007** | **0.859**  **±0.008** | **0.097**  **±0.018** | **0.141**  **±0.008** | **5.708**  **±0.618** | **26.896**  **±1.593** |
|  | Model-2 | 0.826  ±0.013 | 0.698  ±0.011 | 0.789  ±0.016 | 0.150  ±0.041 | 0.211  ±0.008 | 7.430  ±0.709 | 31.462  ±1.631 |
|  | Model-2-pos | 0.528  ±0.005 | 0.787  ±0.007 | 0.855  ±0.006 | 0.097  ±0.013 | 0.145  ±0.006 | 5.726  ±0.394 | 27.061  ±1.446 |
|  | UNet-cls | 0.859  ±0.014 | 0.729  ±0.011 | 0.818  ±0.010 | 0.182  ±0.028 | 0.182  ±0.010 | 8.301  ±1.063 | 34.029  ±2.276 |
|  | UNet | 0.746  ±0.015 | 0.644  ±0.012 | 0.789  ±0.020 | 0.296  ±0.026 | 0.211  ±0.020 | 11.492  ±0.745 | 43.842  ±2.090 |
|  | UNet-pos | 0.495  ±0.005 | 0.726  ±0.007 | 0.821  ±0.006 | 0.189  ±0.017 | 0.179  ±0.006 | 8.159  ±0.803 | 33.603  ±1.601 |
| ST-test | Our model | **0.890**  **±0.002** | **0.830**  **±0.004** | **0.906**  **±0.005** | **0.096**  **±0.006** | **0.094**  **±0.005** | **4.189**  **±0.121** | **21.071**  **±0.610** |
|  | Model-2 | 0.831  ±0.012 | 0.780  ±0.013 | 0.905  ±0.008 | 0.179  ±0.032 | 0.095  ±0.008 | 5.570  ±0.371 | 26.029  ±1.665 |
|  | Model-2-pos | 0.888  ±0.002 | 0.827  ±0.003 | 0.905  ±0.003 | 0.098  ±0.006 | 0.095  ±0.003 | 4.321  ±0.119 | 21.705  ±0.841 |
|  | UNet-cls | 0.872  ±0.006 | 0.817  ±0.007 | 0.906  ±0.004 | 0.118  ±0.016 | 0.094  ±0.004 | 4.482  ±0.175 | 22.243  ±1.120 |
|  | UNet | 0.825  ±0.011 | 0.747  ±0.013 | 0.870  ±0.018 | 0.188  ±0.035 | 0.130  ±0.018 | 7.115  ±0.489 | 32.705  ±2.459 |
|  | UNet-pos | 0.866  ±0.003 | 0.809  ±0.003 | 0.904  ±0.006 | 0.125  ±0.005 | 0.095  ±0.005 | 4.771  ±0.153 | 24.069  ±1.025 |

# More examples of the use simulation of our model on routine BUS screening

Figure S1 – Figure S5 show 5 malignant breast cases, Figure S6 – Figure S10 show 5 benign breast cases. The first row presents the original images extracted from a video recorded during a BUS scan. The second and third rows are segmentation results given by Model-2-pos (corresponding to the red line in Figure 4) and our model, respectively. The marked regions indicate the predicted tumors. The corresponding videos can be viewed and downloaded at <https://www.doi.org/10.6084/m9.figshare.19105910>.

**
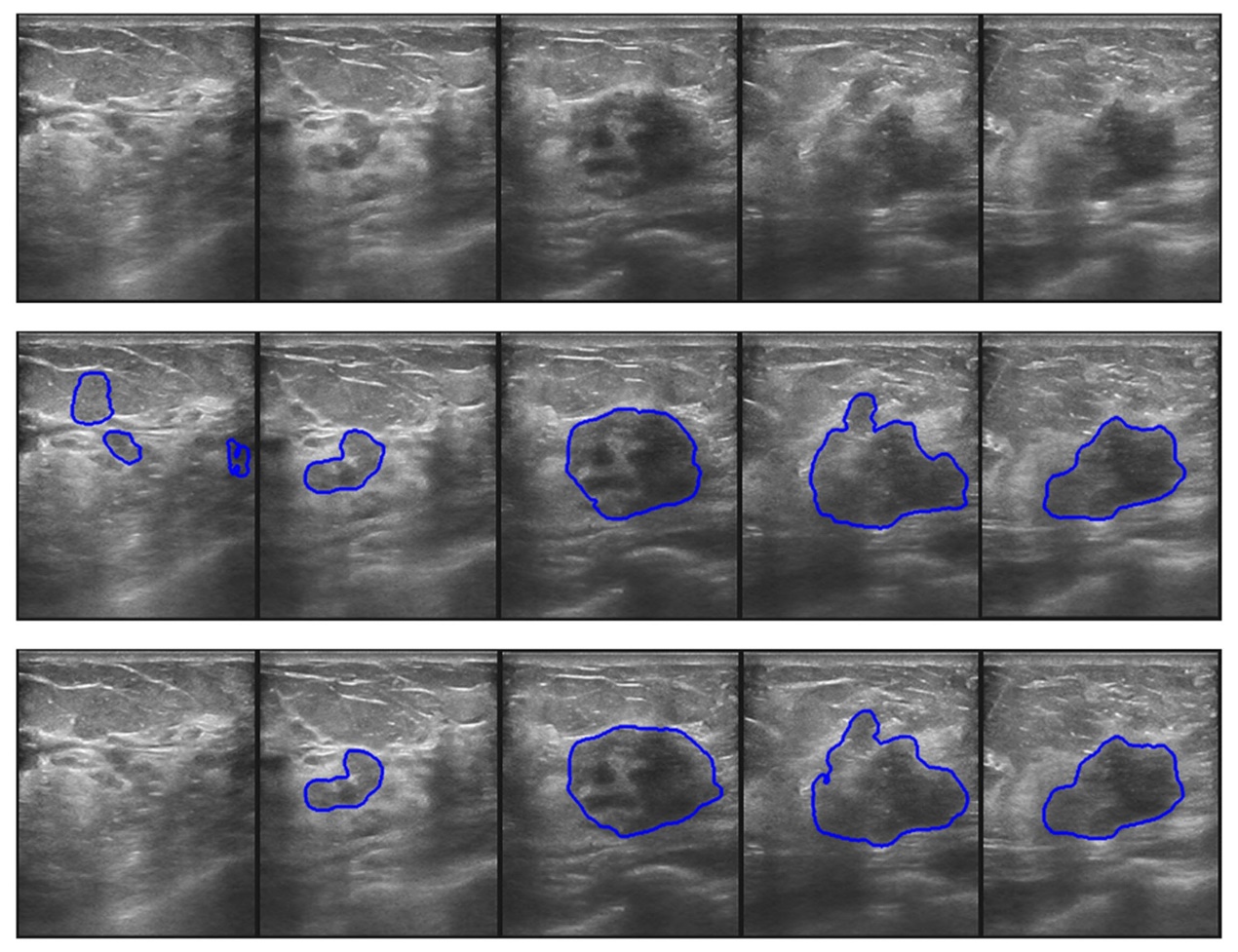
**

Figure S1. Segmentation results for a breast with intraductal carcinoma (malignant case). From left to right, the images were sequentially extracted from a video recorded during a BUS scan. The first, second, third rows present the original images, the tumor regions predicted by Model-2-pos, and the tumor regions predicted by our model, respectively.


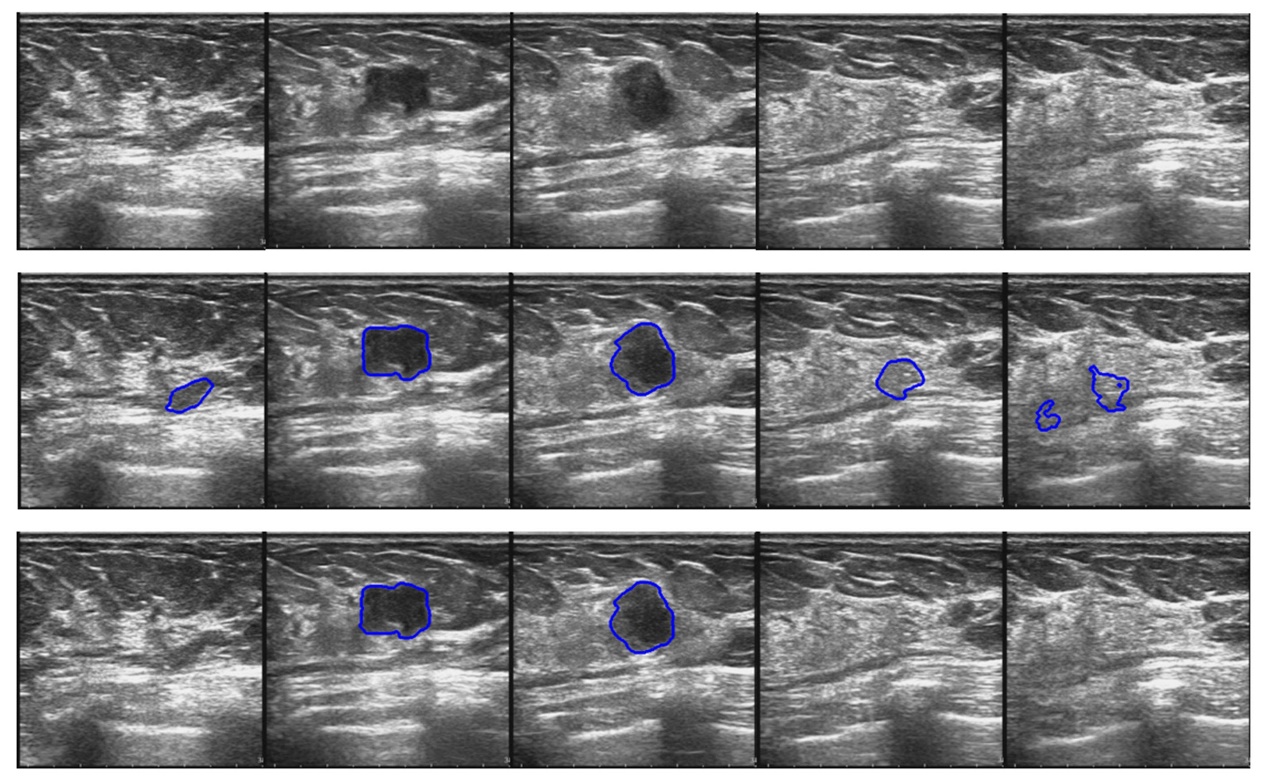


Figure S2. Similar to Figure S1 but for a breast with invasive carcinoma (malignant case).


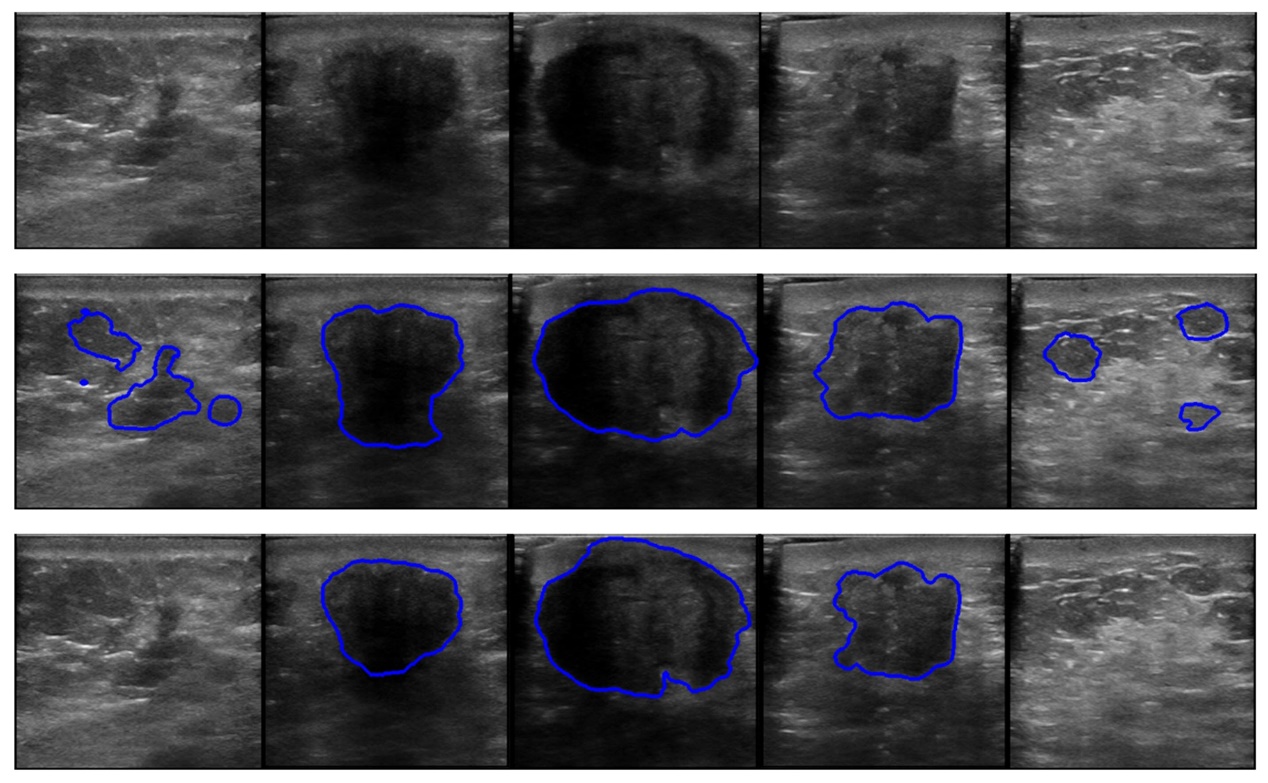


Figure S3. Similar to Figure S1 but for a breast with invasive carcinoma (malignant case).


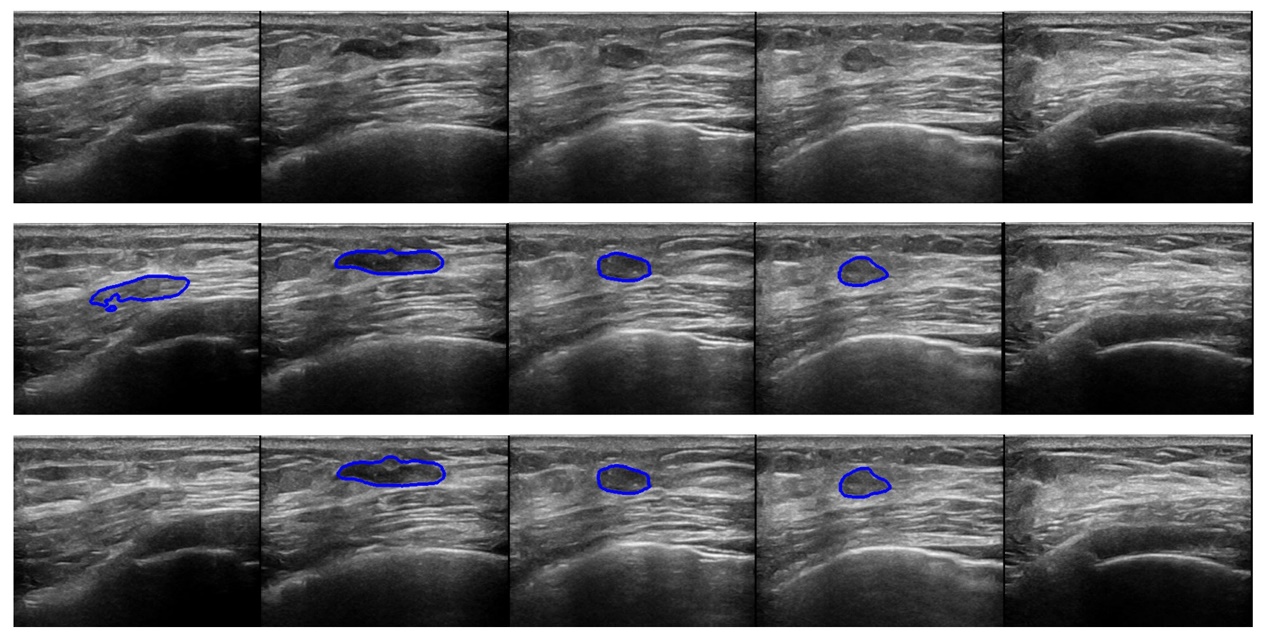


Figure S4. Similar to Figure S1 but for a breast with invasive carcinoma (malignant case).


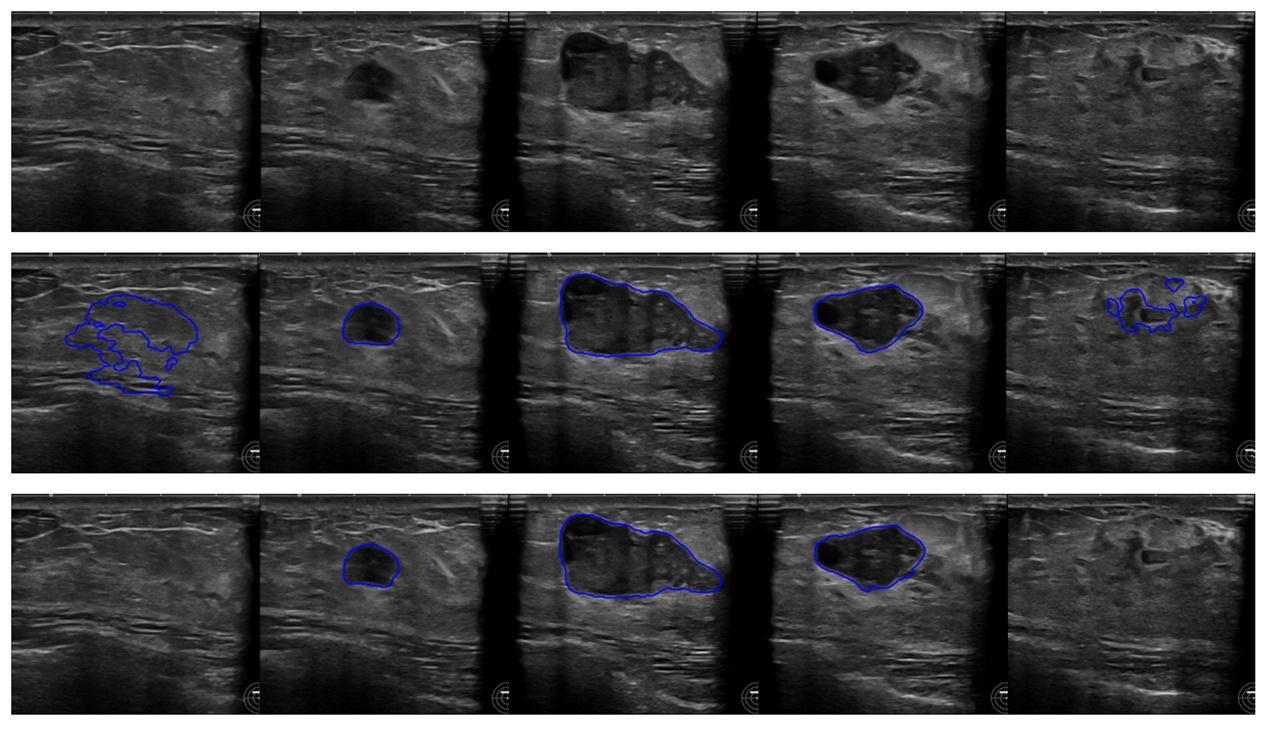


Figure S5. Similar to Figure S1 but for a breast with invasive carcinoma (malignant case).


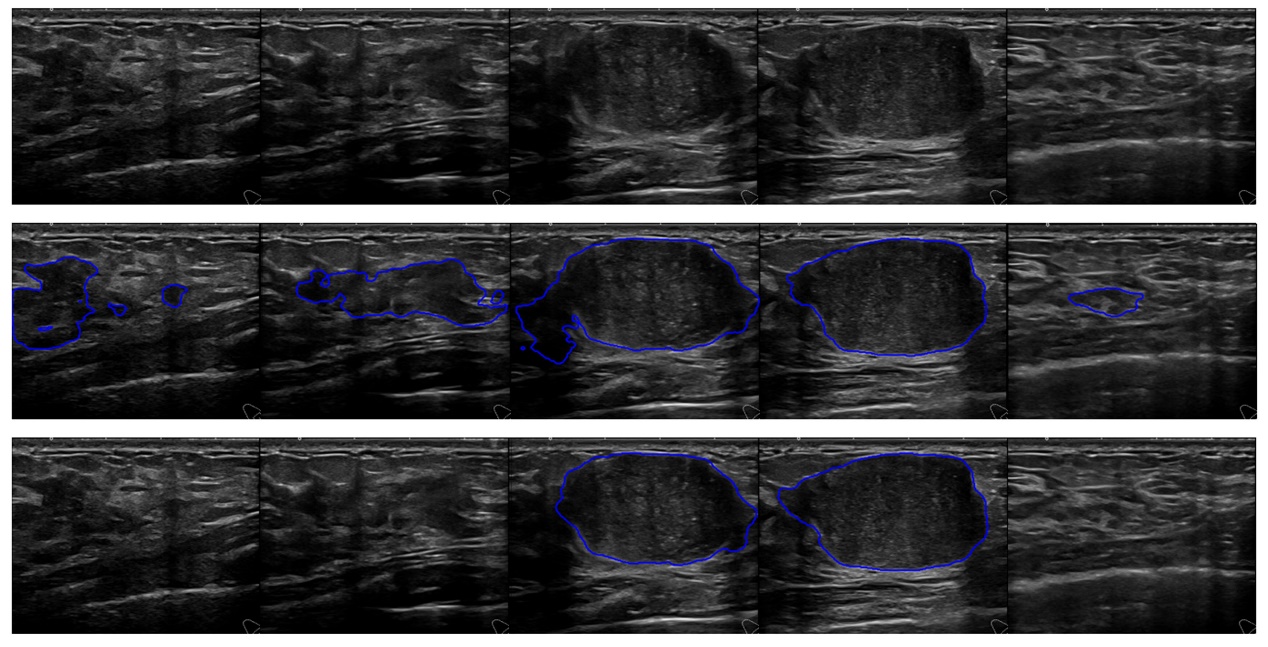


Figure S6. Similar to Figure S1 but for a breast with fibroadenoma (benign case).


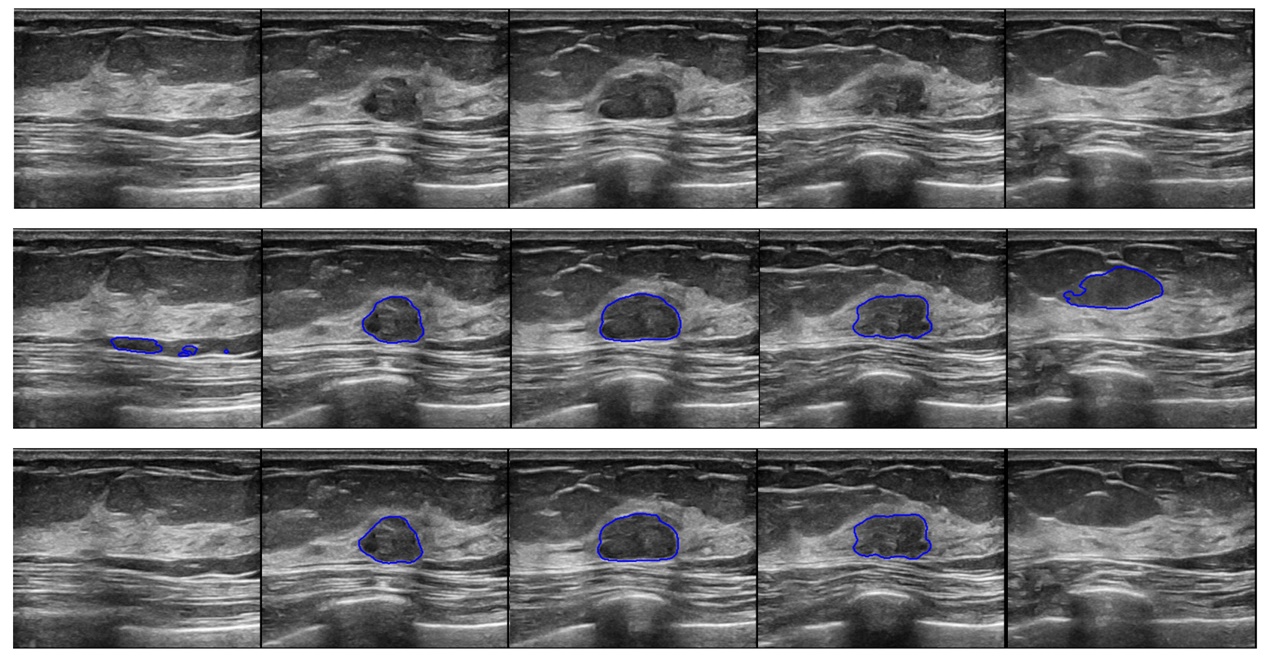


Figure S7. Similar to Figure S1 but for a breast with fibroadenoma (benign case).


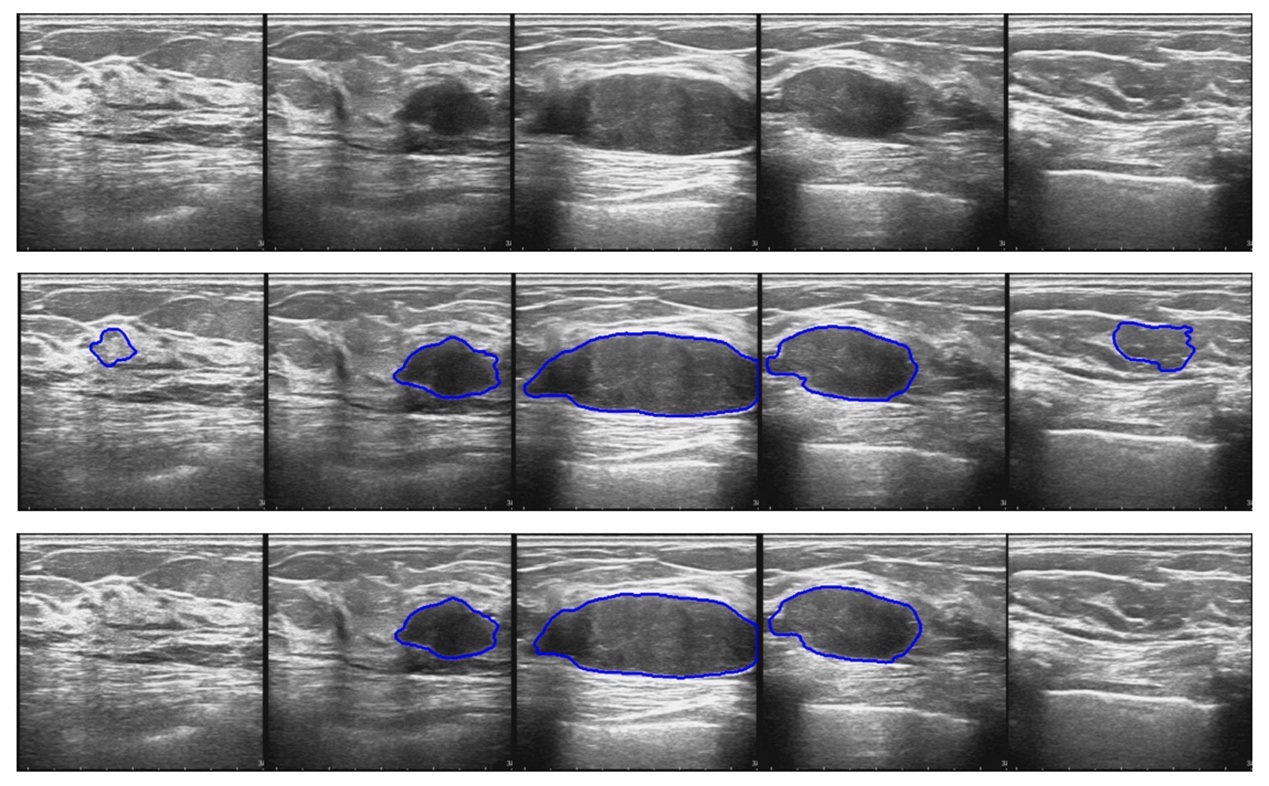


Figure S8. Similar to Figure S1 but for a breast with fibroadenoma (benign case).


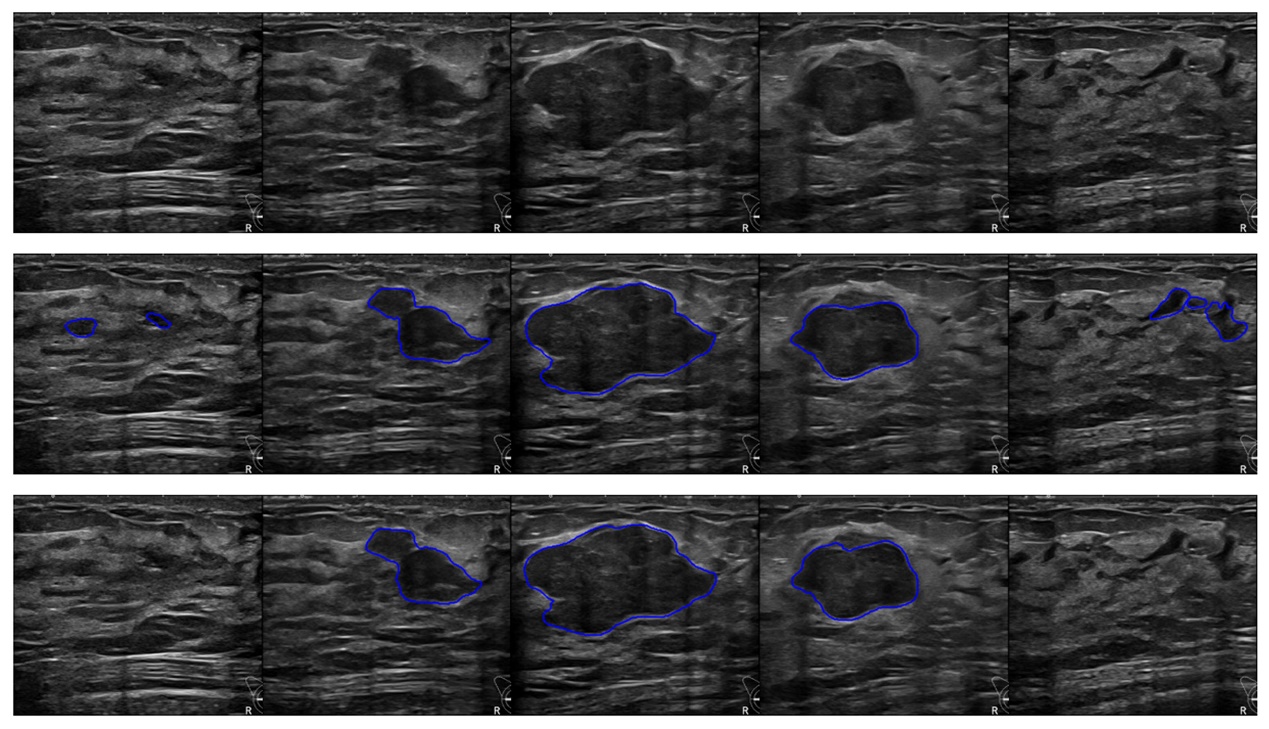


Figure S9. Similar to Figure S1 but for a breast with fibroadenoma (benign case).


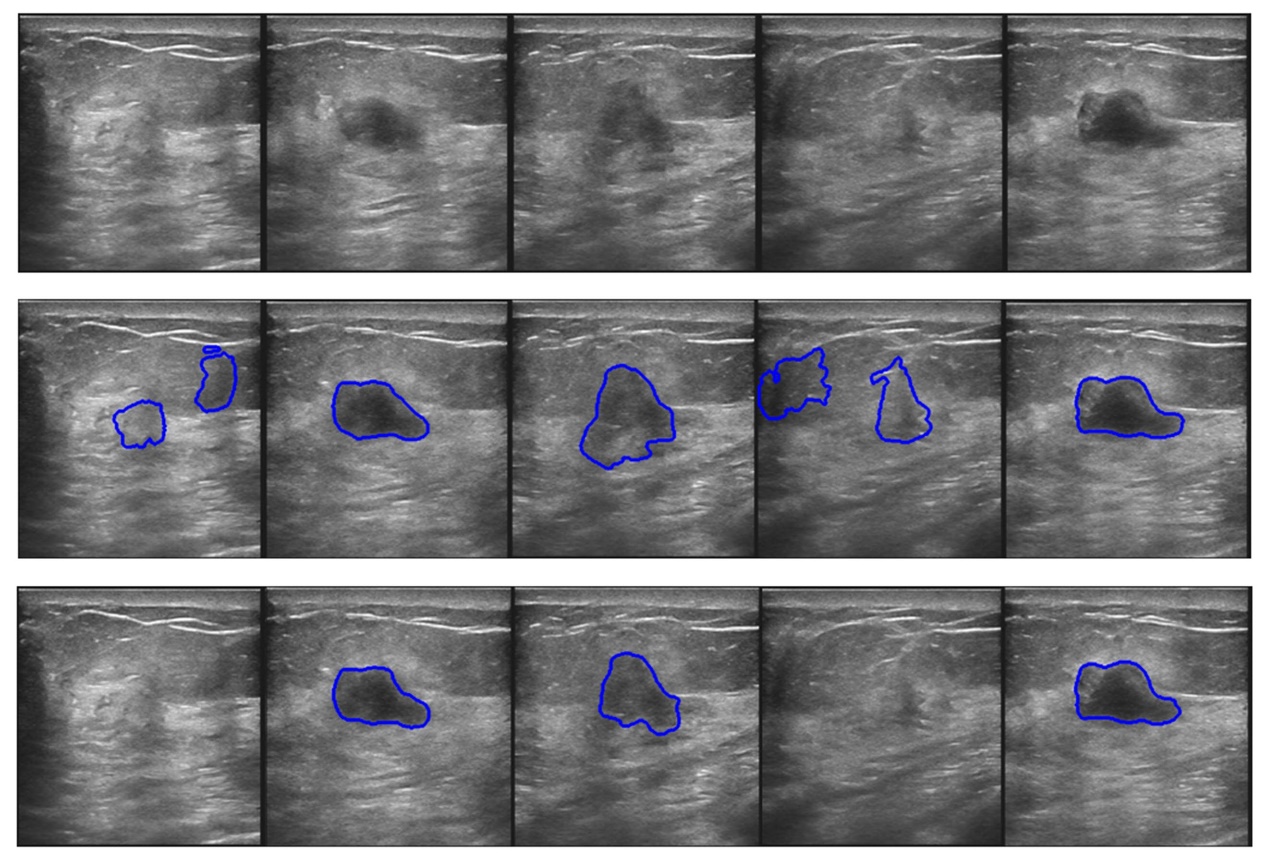


Figure S10. Similar to Figure S1 but for a breast with intraductal papilloma (benign case).
